# Alkylglycerone phosphate synthase (AGPS) deficient mice: Models for rhizomelic chondrodysplasia punctata type 3 (RCDP3) malformation syndrome

**DOI:** 10.1016/j.ymgmr.2014.06.003

**Published:** 2014-08-07

**Authors:** Ryan P. Liegel, Adam Ronchetti, D.J. Sidjanin

**Affiliations:** a Department of Cell Biology, Neurobiology, and Anatomy, Medical College of Wisconsin, Milwaukee, WI, USA; b Human and Molecular Genetics Center, Medical College of Wisconsin, Milwaukee, WI, USA

**Keywords:** RCDP, rhizomelic chondrodysplasia punctata, GNPAT, glyceronephosphate O-acyltransferase, *AGPS*, alkylglycerone phosphate synthase, PTS, peroxisomal targeting signal, KOMP, Knockout Mouse Project, PEX7, peroxisomal biogenesis factor 7, Mutation, AGPS, RCDP3, Mouse, Phenotype, Hypomorphic

## Abstract

Rhizomelic chondrodysplasia punctata (RCDP) is a genetically heterogeneous autosomal recessive syndrome characterized by congenital cataracts, shortening of the proximal limbs, neurological abnormalities, seizures, growth delays, and severe intellectual disability. Most RCDP children die in the first decade of life due to respiratory complications. Mutations in alkylglycerone phosphate synthase (*AGPS*) cause RCDP type 3 (RCDP3). We've previously established that cataracts and male infertility in *blind sterile 2* (*bs2*) mice are caused by a spontaneous hypomorphic mutation in *Agps*. As a part of this study, we set out to further explore the *bs2* phenotypes and how they correlate to the clinical presentations of RCDP3 patients. Our results show that ~ 50% *bs2* mice die embryonically and surviving *bs2* mice exhibit growth delays that they overcome by adulthood. The X-ray analysis of adult *bs2* mice revealed significant humeral, but not femoral shortening. Clinical and histological eye evaluations revealed that *bs2* lenses undergo normal development with first opacities developing at P21 that by P28 rapidly progress to mature cataracts. Evaluation of testes determined that infertility in *bs2* mice is due to the aberrant formation of multicellular cellular clusters that undergo apoptosis. Given that the *bs2* locus is a hypomorphic *Agps* mutation, we set out to generate *Agps* knockout mice utilizing the Knockout Mouse Project (KOMP) resource. Our results showed that ~ 85% of *Agps* knock-out mice die embryonically whereas surviving adult *Agps* knock-out mice phenotypically exhibit cataracts and testicular abnormalities similar to those observed in *bs2* mice. Given that the majority of *Agps* knock-out mice die embryonically, this presented a challenge for further analyses of *Agps* deficiency in mouse models. Although not done as a part of this study, *Agps-KOMP* mice or ES cells can be further modified with FLP recombinase to generate mice suitable for subsequent matings with a transgenic *Cre* strain of choice, thereby providing an opportunity to study conditional *Agps* deficiency in a specific tissue or desired developmental time points without *Agps* deficiency-mediated embryonic lethality.

## 1 Introduction

Rhizomelic chondrodysplasia punctata (RCDP) is a malformation syndrome characterized by bilateral congenital cataracts, severe shortening of the humerus and femur, stippled epiphyses, vertebral coronal clefts, microcephaly, myelination defects, growth and developmental delays, and severe intellectual disability [1], [2]. Only about 50% of RCDP children survive up to 6 years of age and less than 10% reach teenage years [3]. Genetic studies have determined that RCDP is a heterogeneous disorder with three distinct forms: RCDP type 1 (RCDP1) [OMIM 215100], RCDP type 2 (RCDP2) [OMIM 222765], and RCDP type 3 (RCDP3) [OMIM 600121] [1], [4], [5]. RCDP1 is caused by mutations in *PEX7*[6], [7], [8], RCDP2 is caused by mutations in glyceronephosphate O-acyltransferase (*GNPAT*) [9], [10], and RCDP3 is caused by mutations in alkylglycerone phosphate synthase (*AGPS*) [11], [12]. Although genetically distinct, biochemical studies have determined that the depletion of plasmalogens causes all three forms of RCDP [13] and as such all three RCDP forms are clinically indistinguishable.

Plasmalogens are a class of ether lipids where acylation of dihydroxyacetone phosphate at the *sn*-1 position is mediated by the GNPAT enzyme followed by the exchange of the acyl group for an alkyl group by the AGPS enzyme [13], [14]. It has been shown previously that catalytically active AGPS promotes the catalytic activity of GNPAT [15]. Therefore, functional loss of either GNPAT or AGPS disrupts plasmalogen synthesis and results in plasmalogen deficiency in RCDP2 and RCDP3. RCDP1, however, is caused by the functional loss of PEX7, a protein which is the peroxisomal targeting signal 2 (PTS2) receptor mediating import of proteins containing the PTS2 consensus sequence into peroxisomes [16]. Although the majority of peroxisomal proteins contain the PTS1 consensus sequence and are imported into peroxisomes via the PTS1/PEX5 receptor mechanism, AGPS, phytanoyl-CoA hydroxylase, and 3-ketoacyl thiolase contain the PTS2 signal and are imported into the peroxisomes via PTS2/PEX7 receptor mechanism [5]. While PTS2 disruption affects all three proteins, it has been established that plasmalogen deficiency in *RCDP1* patients is caused by the failure of AGPS peroxisomal import, and the consequent AGPS functional loss during synthesis of plasmalogens [6], [17].

How plasmalogen deficiency results in RCDP clinical phenotypes is largely unknown. RCDP mouse models provide an excellent resource for addressing this question. *Pex7^−/−^* and *Gnpat^−/−^* mice exhibit plasmalogen deficiency as well as skeletal, testicular, brain, and eye abnormalities, recapitulating some phenotypes observed in RCDP patients [18], [19]. Recently, our lab showed that *blind sterile 2* (*bs2*) mice exhibiting cataracts and male infertility [20] are caused by a spontaneous mutation in *Agps*[21]. Our analysis identified a G to A substitution at the + 5 position of *Agps* intron 14 that alters splicing resulting in an *Agps*^∆*exon14*^ transcript lacking exon 14, an additional aberrant *Agps*^*exon*∆*13–14*^ transcript lacking both exons 13 and 14, and residual levels of the full-length *Agps* transcript [21]. Both aberrant *Agps*^∆*exon14*^ and *Agps*^*exon*∆*13–14*^ transcripts encode putative truncated catalytically inactive AGPS proteins whereas residual levels of the full-length *Agps* encode putative full-length catalytically active AGPS protein, but at severely reduced levels of about 15% of that observed in WT mice. Mass spectrometry analysis of lipid species from *bs2* confirmed severely reduced levels of plasmalogens; therefore, the *bs2* mouse was established as a hypomorphic *Agps* mutation [21].

As a part of this study, we focused on further evaluation of the *bs2* mouse phenotypes. Our results showed that about half of the *bs2* mice die embryonically and the surviving *bs2* mice exhibit delayed growth, shortening of the humerus, cataracts, and male infertility associated with seminiferous tubule abnormalities. We also set out to create *Agps* knock-out mice utilizing resources from the Knockout Mouse Project (KOMP) [22]. We show that ~ 85% of *Agps* knock-out mice die embryonically which has hindered detailed studies of phenotypes associated with *Agps* deficiency. However, we recovered few adult *Agps* knock-out mice and our analysis showed that phenotypically these mice exhibit growth delays, cataracts, and testicular abnormalities similar to those identified in the *bs2* mice.

## 2 Materials and methods

### 2.1 Mice and genotyping

*bs2*, B6.FVB-Tg(EIIa-*Cre*)C5379Lmgd/J (referred to in the text as ElIa-*Cre*) and C57BL/6J mice were obtained from the Jackson Laboratory (Bar Harbor, ME). Genotyping of the *bs2*, *rd8*, and EIIa-*Cre* alleles was done as described previously [21], [23], [24] using primers summarized in Table 1S. The *bs2* mice were maintained on C57BL/6J × CastEi/J mixed F2 background as previously described by brother to sister breedings [21]. Mice heterozygous for the *Agps^tm1a(KOMP)Wtsi^* allele (referred to in the text as *Agps-KOMP*) were obtained from the Knockout Mouse Project (KOMP) [22] repository at the University of California, Davis. The *Agps-KOMP* allele was genotyped utilizing primers summarized in Table 1S. All primers were synthesized by Integrated DNA Technologies (Iowa City, IA), and used with Platinum *Taq* polymerase (Invitrogen).

### 2.2 Clinical examination

Weights of WT (n = 8) and *bs2* (n = 8) postnatal mice were measured and recorded in littermates from *bs2/+* × *bs2/+* crosses between P0.5 and 4 months of age. Age-matched *bs2* (n = 4), *AGPS-KOMP* mice (n = 4), *Agps-KOMP* EIIa-*Cre* (n = 2) and control (n = 4) mice were X-ray imaged at 4 months of age. Exposures were recorded at a peak kilovoltage of 50 kVp and a charge of 0.50 mAs (milliampere seconds). The same mice were also evaluated with a Topcon SL-D8Z slit lamp biomicroscope with a Nikon SLR-based Photo Slit Lamp imaging system following mydriasis with 1% Atropine Sulfate (Bausch & Lomb). WT (n = 6) and *bs2* (n = 6) testes weights were measured in age-matched pairs between 4 and 8 weeks of age. Significance for all measurements was calculated via two-tailed *t*-test (GraphPad), where *P* < 0.05 was considered significant.

### 2.3 Histology and immunohistochemistry

All tissues were collected, paraffin embedded, and H&E stained as previously described [25]. For evaluations of cataracts, eyes from age-matched *bs2* and control mice were collected at P0.5 (n = 2), P5 (n = 2), P14 (n = 2), P21 (n = 2), P28 (n = 2) and 4 months of age (n = 2) as well *AGPS-KOMP* mice (n = 2), *Agps-KOMP* EIIa-*Cre* (n = 2) and control (n = 2) mice were collected at 4 months of age. For immunohistochemistry, we used antibodies: E-cadherin (Cell Signaling), MIP (Milipore), DAZL (Abcam), and TRA54 (B-Bridge) as primary antibodies and DyLight 488 goat anti-rat or goat anti-rabbit (Abcam) as secondary antibodies following manufacturers' recommendations. Peanut agglutinin lectin (PNA) staining was done with the Lectin PNA-Alexa-488 conjugate (Life Technologies) according to the manufacturers' recommendations. For proliferation studies, EdU was injected intraperitoneally at a concentration of 100 mg/kg 3 h prior to euthanizing the mice; EdU detection was performed with the Click iT EdU Alexa Fluor 488 Imaging Kit and counterstained with DAPI (Life Technologies) according to the manufacturer's recommendations. TUNEL staining was performed utilizing the Chemicon ApopTag Plus In Situ Apoptosis Fluorescein Detection Kit (Millipore) according to the manufacturer's recommendations. All slides were mounted with Vectashield and imaged with a Nikon DS-Fi1 camera on a Nikon Eclipse 80i microscope with NIS-Elements software (Nikon). All cell-counting measurements were performed on sections from a minimum of three separate genotypes with at least ten sections per genotype. Significance was calculated via two tailed *t*-test (GraphPad), where *P* < 0.05 was considered significant.

### 2.4 RT-PCR

Lenses from eyes of WT P5, P14, P21, and P28 mice were collected for RNA analysis. Spleen, kidney, liver, and testes were collected from *Agps-KOMP*, *Agps-KOMP* EIIa-*Cre*, and control mice at 4 months of age. RNA was isolated and reverse transcribed as previously described [21]. Subsequent PCR products were generated using primers in Table 1S, electrophoresed, gel-purified, and sequenced as previously described [25]. Comparative sequence analysis was performed using DNAStar software. For semi-quantitative analysis of *Agps* transcript levels, RT-PCR products were generated while in the exponential phase of PCR amplification using *Gapdh* as an internal control as previously described [21]. PCR band intensities were quantified using ImageJ software (http://rsbweb.nih.gov/ij/) and are expressed relative to *Gapdh*. The results represent at least three independent experiments performed in triplicates. Comparison between genotype groups was analyzed by two-tailed *t*-test and the data is expressed as mean ± SEM. A value of *P* < 0.05 was designated as being statistically significant.

## 3 Results

### 3.1 Growth delays

**Fig. 1:**
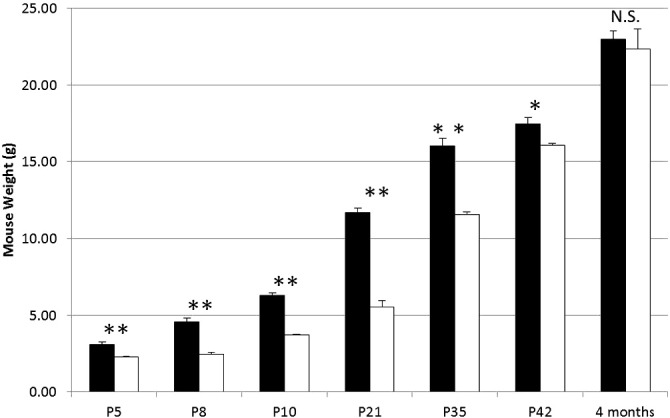
Growth delays in *bs2* mice. Evaluation of the weights of *bs2* (white bars; n = 8) and WT (black bars; n = 8) throughout early postnatal development revealed that *bs2* mice weigh significantly less than WT, but *bs2* catch up to WT littermates by four months of age. Statistical significance was determined by two-tailed *t-*test and error bars represent ± SEM. Double asterisk indicates statistical significance with *P* < 0.001, a single asterisk indicates statistical significance with *P* < 0.05 and N.S. indicates no statistical significance.

At birth, *bs2* pups appeared indistinguishable from WT littermates, but as *bs2* mice progressed through postnatal development, they appeared smaller in size than WT littermates. To further investigate this phenotype, we measured postnatal weights of *bs2* and WT littermates. At P0.5, the weights of *bs2* pups did not significantly differ from WT (not shown). Between P5 and six weeks of age *bs2* mice weighed significantly less than WT littermates; however, by 4 months of age weights of *bs2* mice no longer significantly differed from WT littermates (Fig. 1).

### 3.2 Shortening of the proximal limb phenotypes

**Fig. 2:**
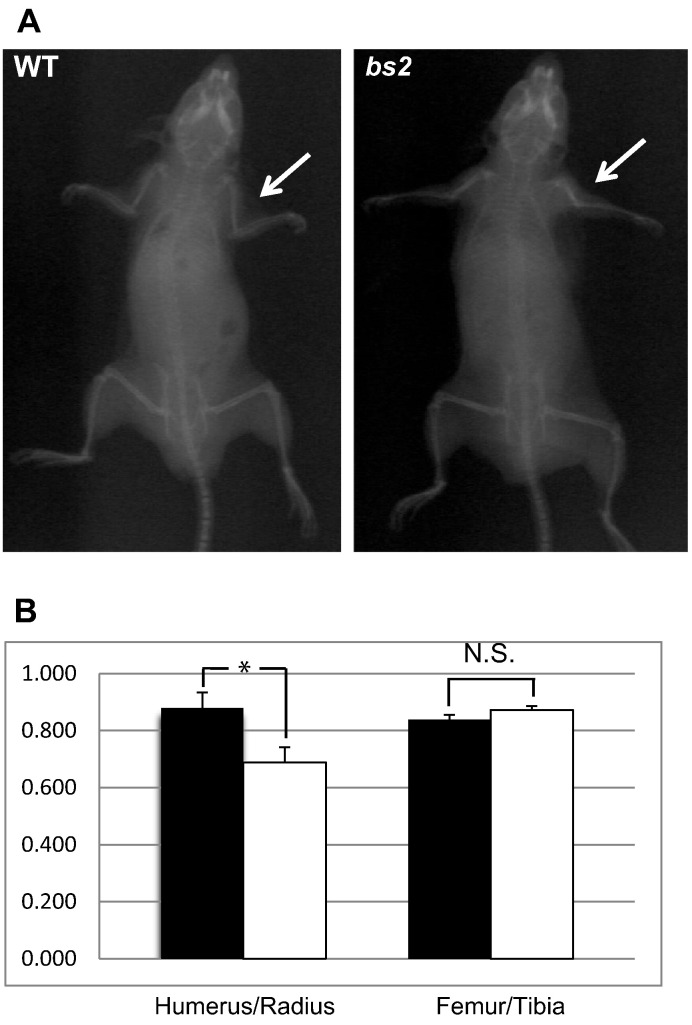
Humeral shortening in *bs2* mice. X-ray imaging (A) of WT and *bs2* mice at 4 months of age reveals shortening of the humerus, indicated by white arrows. As quantified in (B), there is a significant shortening of the humerus/radius ratio in *bs2* (white bar) when compared to WT (black bar) mice (n = 4), while there is no difference in the femur/tibia ratios. Statistical significance was determined by Student's *t* test and error bars represent ± SEM. Asterisk indicates statistical significance with *P* < 0.05 and N.S. indicates no statistical significance.

A profound feature of RCDP patients is the shortening of the proximal limbs [3]. Therefore, we proceeded to evaluate if adult *bs2* mice exhibit shortening of the humerus and femur. X-ray images of 4-month-old WT and *bs2* mice identified shorter humeri, although no shortening of the femur was observed (Fig. 2A). Measurements of the humerus:radius length ratios confirmed a significantly shorter humerus in *bs2* mice whereas no differences were observed in the femurs when comparing femur:tibia ratios of *bs2* to WT (Fig. 2B).

### 3.3 Cataracts

**Fig. 3:**
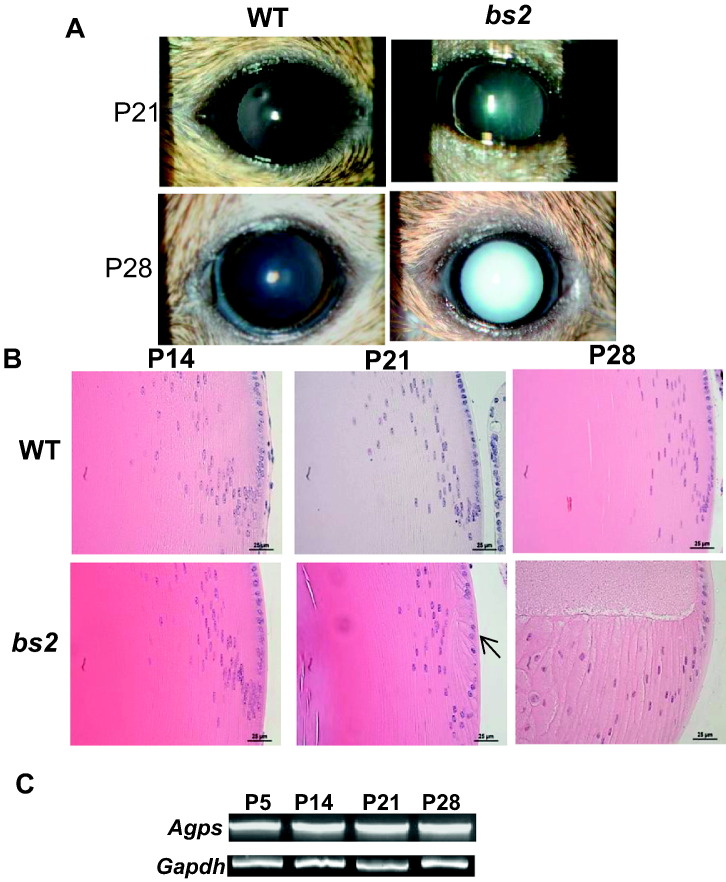
Cataracts in *bs2* mice. (A) Clinical image of mild nuclear cataracts in *bs2* evident at P21 (top right panel) that rapidly progress to mature cataracts by P28 (bottom right panel); no lens abnormalities were noted in WT littermates at P21 (top left panel) or P28 (bottom left panel). (B) Histological analysis prior to P21 did not identify any abnormalities in *bs2* lenses. The initial *bs2* lens abnormalities were observed at P21 characterized by swelling of cells in the transitional zone (black arrow). By P28, *bs2* lenses exhibited detachment of the apical–apical connections between epithelial and fiber cells, as well as swelling and degeneration of fiber cells. Scale bar = 25 μm. (C) RT-PCR analysis using total RNA from postnatal (P) lenses of *Agps* (top panel) and *Gapdh* (bottom panel) revealed expression of *Agps* at all postnatal lenses evaluated.

Our previous study identified severe cataracts in *bs2* mice [21]. Congenital cataracts are a clinical feature in RCDP3 children [15]. Therefore, we focused on determining the onset and progression of the *bs2* cataracts. A slit-lamp evaluation of *bs2* lenses starting at P14 did not identify any opacities prior to P21 (not shown); however at P21 *bs2* lenses exhibited mild nuclear opacities that rapidly progressed to severe total opacities by P28 (Fig. 3A). At later time points (12–24 months), the *bs2* cataracts did not differ from those observed at P28 (not shown). Histological evaluation of *bs2* eyes supported the clinical findings; postnatal eyes (P0.5, P5, P14) did not identify any *bs2* morphological abnormalities (not shown). At P21 the initial abnormality noted was the swelling of the cells in the bow region and by P28, *bs2* lens fiber cells were disorganized, swollen, and detached from their apical–apical connections with lens epithelial cells (Fig. 3B). Immunostaining for E-cadherin (epithelial cells), and MIP (lens fiber cells) in WT and *bs2* lenses prior to the onset of cataracts did not identify any difference between WT and *bs2* mice at P14 (not shown), indicating that lens epithelial cells were undergoing normal development and differentiation. To determine if the cataract onset in *bs2* mice was associated with the temporal expression of *Agps* in the lens, we performed RT-PCR from RNA collected from postnatal WT lenses. Our results identified that *Agps* is expressed throughout postnatal development (Fig. 3C). These findings indicated that in mice *Agps* is not required for lens development, but is required for lens homeostasis.

### 3.4 Testicular abnormalities

**Fig. 4:**
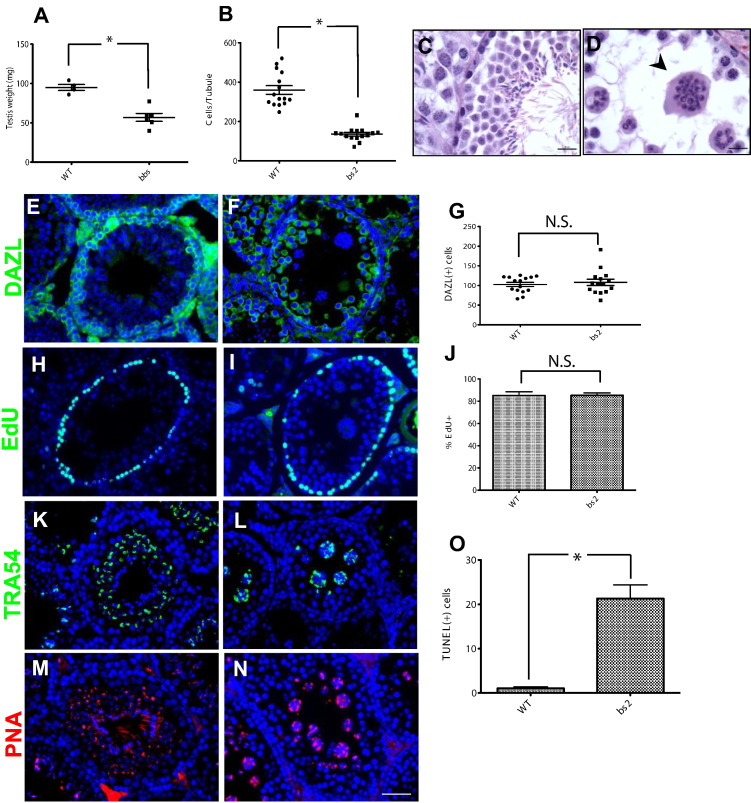
Testicular abnormalities in *bs2* mice. (A) Testes from *bs2* mice (n = 6) weigh significantly less than testes from age-matched WT (n = 6) siblings between 4 and 8 weeks of age, *P* < 0.05. (B) The number of cells per seminiferous tubule in *bs2* was significantly less than the number of cells observed in WT tubules (n = 15). Statistical significance was determined by two tailed *t*-test and error bars represent ± SEM. An asterisk indicates statistical significance with *P* < 0.05. Histological analysis of adult *bs2* seminiferous tubules (D) identified significant germ cell depletion and the presence of large multicellular clusters (arrowhead) that were absent in WT tubules (C). Immunostaining for DAZL, a marker for spermatogonia and Sertoli cells, revealed disorganized DAZL-positive cells in *bs2* tubules (F) in contrast to highly organized DAZL-positive cells in WT tubules (E) even though the number of DAZL-positive cells did not significantly differ between the two genotypes (n = 15) shown in (G). The number of EdU-positive cells in WT (H) and *bs2* (I) tubules did not differ between the two genotypes (n = 15) shown in (J). In *bs2* tubules, TRA54 positive cells were identified within the multicellular clusters (L), whereas in WT tubules characteristic TRA54 crescent shaped staining of spermatids was evident (K). In *bs2* tubules, PNA-positive cells were identified in the multicellular clusters (N), whereas in WT tubules PNA stained spermatids and spermatozoa (M). A significantly greater number of TUNEL-positive cells was evident in *bs2* tubules compared to WT tubules (n = 15) (O). Scale bar = 25 μm.

Our previously published data identified that *bs2* males exhibit smaller testes characterized by the absence of elongated spermatids and spermatozoa [21]. Thus, we proceeded to further characterize *bs2* male testes. The weights of *bs2* adult testes were significantly smaller when compared to the WT controls (Fig. 4A). In addition, the *bs2* seminiferous tubules contained significantly fewer cells (Fig. 4B). Large multicellular clusters were present in *bs2* (Fig. 4D) that were not present in WT tubules (Fig. 4C). To evaluate this phenotype further, we immunostained WT and *bs2* tubules for deleted in azoospermia-like (DAZL), a marker for spermatogonia and Sertoli cells [26]. Our analysis did not identify any significant difference in the number of DAZL-positive cells between the two genotypes (Fig. 4G) although in *bs2* tubules DAZL-positive cells appeared disorganized (Fig. 4F) in contrast to highly organized DAZL-positive cells in WT tubules (Fig. 4E). Next, we focused on the evaluation of the number of proliferating spermatogonia in *bs2* and WT testes. The percentage of EdU positive spermatogonia in WT (Fig. 4H) and *bs2* (Fig. 4I) mice did not significantly differ (Fig. 4J). Immunostaining of WT tubules with TRA54, which is a haploid sperm cell-specific antigen, revealed a punctate staining characteristic of spermatocytes [27] (not shown) and crescent-shaped staining pattern marking the round spermatids [27] (Fig. 4K). In the *bs2* tubules TRA54 positive punctate and crescent-shaped staining were also present, but only within the multicellular clusters (Fig. 4L). Staining with peanut agglutinin (PNA) as the acrosomal marker [28] revealed the presence of spermatids and spermatozoa in WT (Fig. 4M). PNA positive cells were also present in *bs2* tubules within the multiceIIular clusters (Fig. 4N). A significantly greater number of TUNEL positive cells were present in *bs2* when compared to WT tubules (Fig. 4O), primarily present within the multicellular clusters (not shown).

### 3.5 Generating *Agps* knockout mice

Given that *bs2* mice are hypomorphic for AGPS function, we set out to generate *Agps*-knockout mice and determine phenotypical consequences of complete *Agps* deficiency. Two *Agps-KOMP/+*heterozygote mice were recovered and were phenotypically indistinguishable from WT mice, which is consistent with an autosomal recessive inheritance. *Agps-KOMP/+*mice were generated on the C57BL/6N background [22]; however, it was recently determined that C57BL/6N mice carry a mutation in the *Crb1* gene termed *rd8* causing retinal degeneration [29]. Genotyping of *Agps-KOMP/+*mice confirmed the presence of the *rd8* allele. It was previously shown that *bs2* mice and *RCDP3* patients exhibit ocular defects [15]{Liegel in 2011 #4}, we wanted to eliminate any contribution of the *rd8* allele to the ocular phenotypes associated with the *Agps* deficiency. Thus, we set up selective breedings with C57BL/6J mice and subsequently eliminated the *rd8* allele from the *Agps-KOMP* genetic background.

Het to het breeding of *Agps-KOMP/+*mice recovered 8% (4/51) of homozygote *Agps-KOMP* mice, deviating from the expected Mendelian ratio of 25%. This finding suggested ~ 70% prenatal lethality for homozygote *Agps-KOMP* mice. In addition to the presence of the gene-trapped cassette designed to disrupt *Agps* splicing following exon 1, *loxP* sites flank both sides of *Agps* exon 2 [30]; therefore, crossing of *Agps-KOMP* mice to EIIa-*Cre* mice shown to efficiently remove transcripts from all tissues during early embryonic development [23], would assure that we generated the full global *Agps* knockouts. Breeding of homozygote *Agps-KOMP* females to *Agps-KOMP/+*EIIa-*Cre* males recovered 3.7% (2/53) of *Agps-KOMP* EIIa-*Cre* homozygote mice deviating from the 25% expected Mendelian ratio thereby suggesting ~ 85% prenatal lethality of *Agps-KOMP* EIIa-*Cre* mice.

### 3.6 Analysis of *Agps-KOMP* and *Agps-KOMP* EIIa-*Cre* mice

**Fig. 5:**
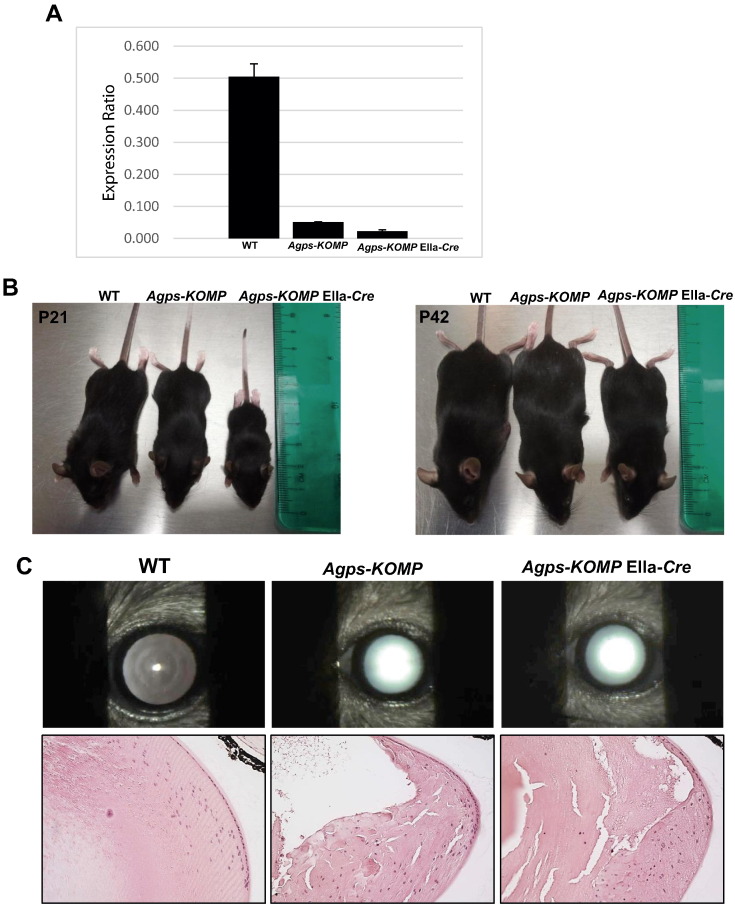

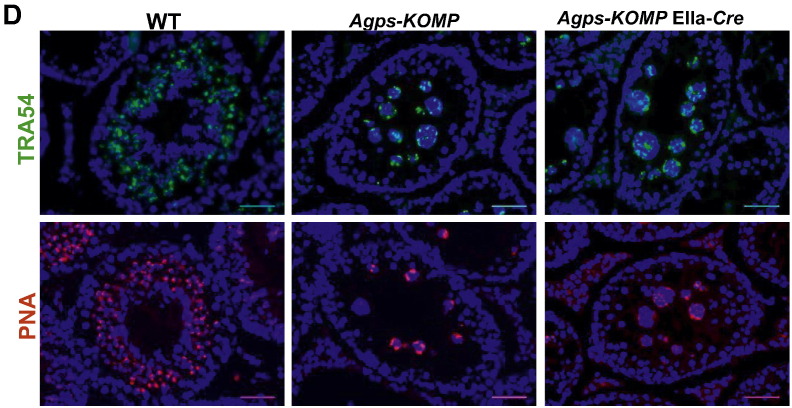
Characterization of *Agps-KOMP* and *Agps-KOMP* Ella-*Cre* mice. Semi-quantitative RT-PCR (A) shows ratios of full-length *Agps* and *Gapdh* transcripts in WT, *Agps-KOMP*, and *Agps-KOMP* Ella-*Cre* testes (n = 3; error bars represent ± SEM). (B) At P21, *Agps-KOMP* mice did not appear to differ in size and *Agps-KOMP* Ella-*Cre* appeared smaller when compared to age-matched WT littermates; however by six weeks *Agps-KOMP* Ella-*Cre* only appeared slightly smaller than age-matched *Agps-KOMP* or WT. Both *Agps-KOMP* and *Agps-KOMP* Ella-*Cre* mice exhibited severe cataracts (C) identified following clinical evaluation (top panel) as well as histological evaluation (bottom panel). Both *Agps-KOMP* and *Agps-KOMP* Ella-*Cre* mice exhibited the presence of multicellular clusters (D) containing TRA54 and PNA positive cells. Scale bar = 25 μm.

Only a few *Agps-KOMP* (n = 4) and *Agps-KOMP* EIIa-*Cre* mice (n = 2) were recovered at birth and these mice survived into adulthood. Semi-quantitative RT-PCR of RNA from *Agps-KOMP* and *Agps-KOMP* EIIa-*Cre* tissues identified residual levels of full-length *Agps* at about ~ 11% and < 5% respectively of the levels observed in WT (Fig. 5A). Throughout the postnatal development, *Agps-KOMP* mice did not appear to differ in size from the WT mice whereas *Agps-KOMP* EIIa-*Cre* mice appeared smaller in size at P21 and by six weeks *Agps-KOMP* EIIa-*Cre* mice appeared only slightly smaller when compared to the WT littermates (Fig. 5B). Slit-lamp evaluation of *Agps-KOMP* and *Agps-KOMP* EIIa-*Cre* eyes prior to P21 did not identify any lens opacities (not shown). At P21, both *Agps-KOMP* and *Agps-KOMP* EIIa-*Cre* mice exhibited mild bilateral nuclear cataracts (not shown) that rapidly progressed to severe cataracts observed at P28 (not shown) that did not progress further by 4 months of age (Fig. 5C). Histological evaluation of the 4-month old *Agps-KOMP* and *Agps-KOMP* EIIa-*Cre* lenses confirmed severely disrupted lens fiber cells (Fig. 5C). The X-ray analysis of *Agps-KOMP* and *Agps-KOMP* EIIa-*Cre* mice did not identify any obvious shortening of either humerus:radius length ratios or femur:tibia length ratios (not shown). *Agps-KOMP* and *Agps-KOMP* EIIa-*Cre* females were able to produce litters whereas *Agps-KOMP* and *Agps-KOMP* EIIa-*Cre* males were infertile. *Agps-KOMP* and *Agps-KOMP* EIIa-*Cre* testes sizes appeared smaller than WT (not shown) and immunohistochemical analysis determined *Agps-KOMP* and *Agps-KOMP* EIIa-*Cre* seminiferous tubules did not differ from WT in the number of DAZL positive cells and in the number of proliferating spermatogonia (not shown). Additionally, the lumen of *Agps-KOMP* and *Agps-KOMP* EIIa-*Cre* tubules contained multicellular clusters of TRA54 and PNA positive cells (Fig. 5D) that were phenotypically indistinguishable from those observed in *bs2* testes (Fig. 4L and N).

### 3.7 Discussion

As a part of this study, we focused on further defining the mouse phenotypes associated with *Agps* deficiency. Our previous study determined that a hypomorphic *Agps* mutation expressing approximately 15% of the full-length *Agps* transcript levels causes cataracts and male infertility in *bs2* mice [21]. As a part of this study, we generated *Agps* knock-out mice utilizing KOMP resources. The *Agps* knockout construct was designed as a “Knockout First Mutation (Reporter Tagged Insertion With Conditional Potential)” predicted to generate a null allele through splicing of exon 1 to a *lacZ* trapping element contained in the targeting cassette that additionally contains the mouse *En2* splice acceptor and the SV40 polyadenylation sequences [30], [31]. This strategy was previously shown to be highly effective in creating null alleles in mice [32]. Our results showed that *Agps-KOMP* is an *Agps* hypomorphic mutation exhibiting ~ 11% of full length *Agps* transcript levels when compared to WT mice. Our findings indicate that *Agps-KOMP* construct is “leaky” allowing for some residual full-length *Agps* transcript expression. The “Knockout First Mutation” construct also contains *loxP* sites flanking *Agps* exon 2 [30], [31]. Therefore, we crossed *Agps-KOMP* to EIIa-*Cre* mice in order to remove *Agps* exon 2 and generate a knock-out. In EIIa-*Cre* mice, the *Cre* transgene is under the control of the adenovirus EIIa promoter that targets expression of CRE recombinase to the early mouse embryo prior to implantation in the uterine wall [23]. The presence of less than 5% of full-length *Agps* transcripts in *Agps-KOMP* EIIa-*Cre* mice is most likely associated with the previously reported mosaic expression of the EIIa-*Cre* transgene where efficiency of the *EIIa-Cre* allele was determined to be dependent on the target gene and on parental transmission of the transgene [33].

Our results showed that *Agps-KOMP* and *Agps-KOMP* EIIa-*Cre* mice exhibited ~ 70% and ~ 85% embryonic lethality respectively. We previously reported that *bs2* mice exhibited ~ 50% prenatal lethality [21]. These findings indicate that levels of full-length *Agps* expression strongly correlate to the embryonic lethality in the *Agps* deficient mice. The embryonic lethality observed in all *Agps-*deficient mice presented an obstacle for detailed evaluation of *Agps* deficiency in a mouse model. However, *Agps-KOMP* mice or ES cells can be further modified with FLP recombinase to remove gene-trap cassette mediating “Knockout First Mutation” thus generating a WT allele with *loxP* sites on either side of *Agps* exon 2 [30]. This would allow for a subsequent cross with a transgenic *Cre* strain of choice, thereby providing an opportunity to study *Agps* deficiency in a specific tissue or desired developmental time points without *Agps* deficiency-mediated embryonic lethality.

The cause of embryonic lethality in *Agps*-deficient mice is unknown. Embryonic lethality was also reported for *Gnpat^−/−^* mice [19], although *Pex7^−/−^* only exhibited postnatal lethality [17], [34]. The surviving *bs2* mice were smaller throughout development when compared to WT littermates, a phenotype that *bs2* were able to overcome later on in the development. Although we recovered a very small number of *Agps-KOMP* and *Agps-KOMP* EIIa-*Cre* suitable for a statistical analysis, our observation suggested that adult *Agps-KOMP* mice did not differ in size from age-matched WT littermates whereas the surviving *Agps-KOMP* EIIa-*Cre* mice similar to *bs2* mice exhibited growth delay that they overcame by adulthood. The cause of the growth delay in *bs2* and *Agps-KOMP* EIIa-*Cre* mice also remains unknown. Most RCDP children die during the first decade of life due to the complications associated with pulmonary hypoplasia [3], [35]. It is possible that prenatal death of the *Agps* deficient mice, as well as growth delays observed in the *bs2* and *Agps-KOMP* EIIa-*Cre* mice are associated with a defect and/or delay in the lung development. However, these studies would require further evaluation.

Proximal shortening of the humerus, and to a lesser extent of the femur, is one of the distinctive characteristics in all RCDP patients [3], [36]. Our results identified a shortening of the humerus in the *bs2* mice. Although a very few *Agps-KOMP* and *Agps-KOMP* EIIa-*Cre* mice were evaluated for humeral/femoral phenotypes, our analysis did not identify any obvious rhizomelic bone shortening in these mice. This difference in the humeral phenotype between *bs2*, *Agps-KOMP* and *Agps-KOMP* EIIa-*Cre* mice may be associated with a different genetic background of *bs2* and *Agps*-*KOMP* mice. This is further supported by the observation that shortening of the proximal limbs was reported in the *Gnpat^−/−^* mice [19], but not in *Pex7^−/−^* mice, although *Pex7^−/−^* mice exhibited a defect in ossification in the distal bone elements of the limbs, skull and vertebrae [18]. These findings suggest that in mice plasmalogen deficiency adversely affects skeletal development, where the severity of skeletal abnormalities may also be influenced by genetic modifiers resulting in a range of skeletal phenotypic manifestations.

In all *Agps-*deficient mice from this study, the lenses underwent normal development up to P21. The initial lens abnormality evident at P21 was characterized by the swelling of epithelial cells in the equatorial region ultimately leading to severe lens fiber cell degeneration. In children with RCDP, bilateral congenital cataracts present at birth or during early infancy [3], [37]. *Gnpat^−/−^* and *Pex^−/−^ 7* mice also exhibit cataracts [18], [19], [34] although the molecular etiology of cataract formation in these mice is unknown. Histological lens analyses from a single RCDP patient with unknown genetic etiology revealed swelling of the equatorial epithelial cells and severe abnormalities of the fiber cells [38], [39]. It was concluded that plasmalogen deficiency in RCDP patients leads to aberrant formation of secondary lens fiber cells and that lens fiber cell abnormalities are not due to a degeneration of already formed lens fibers [38], [39]. Our findings show that in mice plasmalogens are not required for lens development, but they play a critical role in lens homeostasis. This discrepancy between mouse and human cataracts phenotypes associated with plasmalogen deficiency may be because there are significant differences in the lipid composition between human and mouse lenses [40]. Human lenses contain especially high levels of plasmalogens [40], [41], therefore, a plasmalogen deficiency may more adversely affect the human lenses than mouse lenses thus resulting in developmental lens fiber cell abnormalities.

We reported previously that *bs2* mice exhibit male infertility [21]. Consistent with these findings, *Agps-KOMP* and *Agps-KOMP* EIIa-*Cre* male mice are also infertile and exhibit testicular abnormalities phenotypically indistinguishable from *bs2* mice. It was shown previously in *Gnpat^−/−^* mice that azoospermia is due to the arrest of spermatogenesis at the level of round spermatids [19]. Additionally, it was shown that plasmalogen deficiency in *Gnpat^−/−^* mice has adverse effects on the establishment and remodeling of the blood–testis barrier, thereby providing evidence that plasmalogens regulate blood–testis permeability and the endocytosis of junctional complexes [42]. Our results support the previously described role of plasmalogens in the development and/or remodeling of the blood–testis barrier [42]. *Agps* deficient mice don't exhibit abnormalities in germ cell proliferation and maintenance and our results also provide evidence that spermatogenesis in *Agps* deficient mice progresses beyond spermatids as indicated by the presence of TRA54 and PNA positive cells in *Agps* deficient tubules. However, they remain “trapped” in aberrantly formed luminal multicellular clusters and undergo apoptosis ultimately resulting in azoospermia and infertility.

### 3.8 Conclusions

Our results show that in mice proper AGPS function is important for embryonic survival, as well as for the normal development of the lens, testes, and humerus. The molecular mechanisms governing the sensitivity of these tissues to *Agps* deficiency remain unclear and require further investigation. Embryonic lethality of *Agps-*deficient mice presents an obstacle for generating a sufficient number of *Agps*-deficient mice suitable for further studies. However, *Agps-KOMP* mice or ES cells can be further modified with FLP recombinase to generate mice suitable for conditional *Agps* ablation with *Cre* strain of choice allowing generation of a sufficient number of conditionally *Agps* ablated mice suitable for detailed morphological and functional analyses.

## Figures and Tables

**Table 1:** A list of primers used in the study.

Primer	Sequence	Purpose
mAgps cDNA 38F	CCGCTCTGAGTGACCTTTCAC	Agps RT-PCR
mAgps cDNA 681R	ATCAGGAATCCGCTCAAACATCC	Agps RT-PCR
mAgps cDNA 390F	GGTTGAGCTGACTGGGAAAA	Agps RT-PCR, Agps semiquantitative RT-PCR
mAgps cDNA 1024R	TCATGCCTGACGCTCGAGTA	Agps RT-PCR, Agps semiquantitative RT-PCR
mAgps cDNA 884F	CAGCTCACGTAGAGGCTGGGATA	Agps RT-PCR
mAgps cDNA 1579R	GCAAATAACCCCTCTGACCGTTA	Agps RT-PCR
mAgps cDNA 1477F	GACCGTGAGAAGGTTCTTCAGCA	Agps RT-PCR
mAgps cDNA 2176R	GCACAGAACAAAAACCAACTCATTT	Agps RT-PCR
mAgps cDNA 1204F	AGACCAACCCCTGAGTACCAGAA	Agps RT-PCR
mAgps cDNA 1768R	TCTGTGTCACCCTGCACGTAGAA	Agps RT-PCR
Gapdh cDNA F	CTTTGGCATTGTGGAAGGG	Gapdh RT-PCR
Gapdh cDNA R	CCTCTCTTGCTGCAGTGTC	Gapdh RT-PCR
mAgps Exon 14F	CTTTCTGCAGGCCAGGCAGTG	bs2 allele
mAgps Exon 14R	CAAATTTTGGAGTAAGAAGAGTTTTT	bs2 allele
Cre-F	GCGGTCTGGCAGTAAAAACTATC	Cre allele
Cre-R	GTGAAACAGCATTGCTGTCACTT	Cre allele
rd8-F	GGTGACCAATCTGTTGACAATCC	rd8 allele
rd8-R	GCCCCATTTGCACACTGATGAC	rd8 allele
Agps KOMP F	GCAGTGGCTCTCAACCTTCCTAAA	Agps KOMP allele
Agps KOMP R1	TGGGAAAGGGTTCGAAGTTCCTA	Agps KOMP allele
Agps KOMP R2	CCAGAGTTCCCAGATCCTACTTCCA	Agps KOMP allele
